# Adenylyl Cyclases 1 and 8 Initiate a Presynaptic Homeostatic Response to Ethanol Treatment

**DOI:** 10.1371/journal.pone.0005697

**Published:** 2009-05-27

**Authors:** Alana C. Conti, James W. Maas, Krista L. Moulder, Xiaoping Jiang, Bhumy A. Dave, Steven Mennerick, Louis J. Muglia

**Affiliations:** 1 Department of Pediatrics, Washington University in St. Louis, St. Louis, Missouri, United States of America; 2 Department of Molecular Biology and Pharmacology, Washington University in St. Louis, St. Louis, Missouri, United States of America; 3 Department of Psychiatry, Washington University in St. Louis, St. Louis, Missouri, United States of America; 4 Department of Anatomy and Neurobiology, Washington University in St. Louis, St. Louis, Missouri, United States of America; National Institutes of Health, United States of America

## Abstract

**Background:**

Although ethanol exerts widespread action in the brain, only recently has progress been made in understanding the specific events occurring at the synapse during ethanol exposure. Mice deficient in the calcium-stimulated adenylyl cyclases, AC1 and AC8 (DKO), demonstrate increased sedation duration and impaired phosphorylation by protein kinase A (PKA) following acute ethanol treatment. While not direct targets for ethanol, we hypothesize that these cyclases initiate a homeostatic presynaptic response by PKA to reactivate neurons from ethanol-mediated inhibition.

**Methodology/Principal Findings:**

Here, we have used phosphoproteomic techniques and identified several presynaptic proteins that are phosphorylated in the brains of wild type mice (WT) after ethanol exposure, including synapsin, a known PKA target. Phosphorylation of synapsins I and II, as well as phosphorylation of non-PKA targets, such as, eukaryotic elongation factor-2 (eEF-2) and dynamin is significantly impaired in the brains of DKO mice. This deficit is primarily driven by AC1, as AC1-deficient, but not AC8-deficient mice also demonstrate significant reductions in phosphorylation of synapsin and eEF-2 in cortical and hippocampal tissues. DKO mice have a reduced pool of functional recycling vesicles and fewer active terminals as measured by FM1-43 uptake compared to WT controls, which may be a contributing factor to the impaired presynaptic response to ethanol treatment.

**Conclusions/Significance:**

These data demonstrate that calcium-stimulated AC-dependent PKA activation in the presynaptic terminal, primarily driven by AC1, is a critical event in the reactivation of neurons following ethanol-induced activity blockade.

## Introduction

Ethanol is a widely used central nervous system depressant that results in sedation. In rodents, the duration of sedation is affected by neuroadaptation to acute ethanol doses; however, the neuroadaptive mechanisms resulting from ethanol exposure remain unclear. The cAMP signaling pathway has emerged as an important modulator of ethanol sensitivity. Reductions in cAMP signaling increase behavioral sensitivity to ethanol in the mouse [1], [2]. We have previously demonstrated that mice lacking the calcium-stimulated adenylyl cyclases 1 and 8 (AC1 and AC8) exhibit increased ethanol-induced sedation compared to controls [1].

AC1 and AC8 generate cAMP from ATP and are the only AC isoforms primarily stimulated by calcium via calmodulin activation [3]–[6]. AC1 and AC8 are expressed in the brain throughout development and adulthood [7]. AC8 localizes to the CA1/CA2 region of the hippocampus, retrosplenial cortex, and thalamus with diffuse expression in the cerebellum and cerebral cortex. AC1 is intensely expressed in hippocampal mossy fiber projections and the cerebellum and at lesser amounts throughout the cortex and thalamus. Subcellular analyses revealed prominent postsynaptic/extrasynaptic expression of AC1, while AC8 localized with presynaptic/extrasynaptic proteins, suggesting that AC1 and AC8 are critical to synaptic events [7] As extrasynaptic protein localization represents both pre- and post-synaptic compartments, it is possible that AC1 can also function presynaptically while AC8 may play a postsynaptic role.

Genetic deletion of AC1 (AC1KO), AC8 (AC8KO) and/or AC1/AC8 (DKO) disrupts long-term depression and potentiation (LTP) [5], [8], [9] as well as late-phase LTP, resulting in memory impairment [6]. Disrupted barrel formation is associated with a loss-of-function mutation in the AC1 gene (*barrelless*). Impaired barrel map development due to reduced AC1-dependent phosphorylation of Rab3-interacting molecule 1α (RIM1α), a PKA target in the presynaptic release apparatus [10], impairs neurotransmitter release from thalamocortical afferents in *barrelless* mice.

Additional data supports cAMP/PKA regulation of presynaptic activity by modulation of exocytotic machinery [11], [12]. PKA recruits synaptic vesicles to the readily releasable vesicle pool, presynaptically regulating synaptic efficacy and plasticity [13]. Furthermore, modulation of depolarization–evoked vesicle exocytosis by PKA phosphorylation of synapsin I is primarily caused by calmodulin-dependent activation of cAMP pathways [14] while calcium/calmodulin-dependent phosphorylation of synapsins I and II regulates vesicle release probability during high-frequency stimulation [15]. Therefore, the synaptic vesicle-associated synapsin phosphoproteins act at the intersection of cAMP and calcium-dependent cascades making them optimal candidates to translate changes in cAMP levels into modulation of vesicle recycling.

We have demonstrated previously that the increased sensitivity of DKO mice to ethanol-induced sedation was accompanied by impaired PKA phosphorylation of target proteins of unknown identity. We hypothesize that ethanol-mediated induction of PKA phosphorylation is part of a compensatory homeostatic mechanism initiated by AC1 and/or AC8. Here, we have used phosphoproteomic techniques and identified several PKA target proteins involved with presynaptic function, including synapsin, vacuolar H+-ATPase, and dynein, that are phosphorylated following acute ethanol exposure in WT mice. Identification of additional proteins phosphorylated after ethanol treatment include dynamin and eukaryotic elongation factor-2 (eEF-2). Of these, we have demonstrated that phosphorylation of synapsin I, II, eEF-2 and dynamin is impaired in the brains of DKO, and in some cases, AC1KO mice following acute ethanol exposure. Together these data suggest that calcium-stimulated ACs, largely involving AC1, contribute to the presynaptic homeostatic response to ethanol-induced inhibition of neuronal function by facilitating PKA activation of proteins involved in presynaptic vesicle release.

## Results

### Targets of ethanol-induced PKA phosphorylation are components of presynaptic vesicle recycling machinery

**Figure 1:**
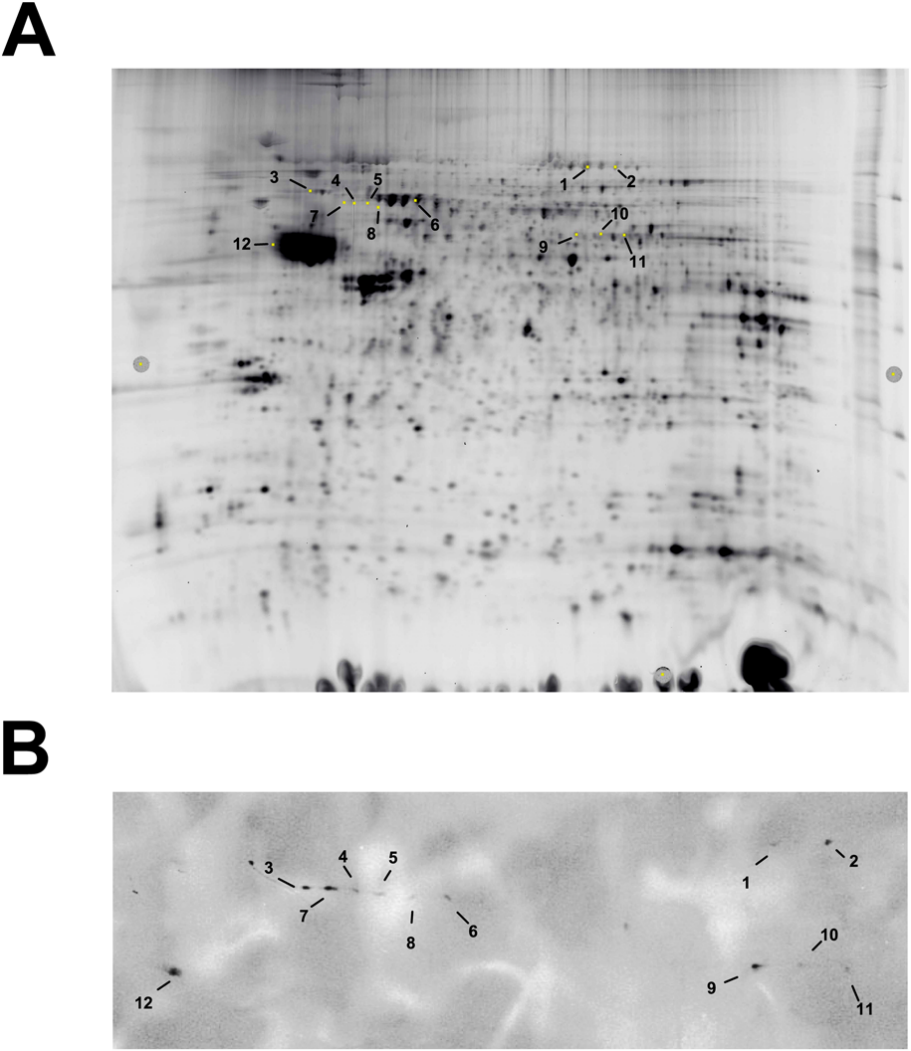
2-Dimensional High Resolution Gel Electrophoresis detects PKA targets phosphorylated following ethanol treatment in WT mice. (A) Protein expression map of cortical lysates from WT mice treated with ethanol separated in two dimensions. (B) Immunoblot of proteins detected using phosphorylated PKA substrate antibody. Spots of interest were excised and processed for MALDI mass spectrometry. Annotations are provided in Table 1.

To identify phosphorylation targets, we performed high-resolution 2-Dimensional Gel Eelectrophoresis, PKA target protein detection using an anti-PKA substrate-specific antibody followed by matrix-assisted laser desorption ionization (MALDI/TOF/TOF) analysis (Figure 1). Analysis of whole cell cortical lysates from WT mice revealed several phospho-protein targets that were phosphorylated within 45 min following acute ethanol exposure, just prior to WT mice awakening from ethanol-induced sedation. Identified proteins included: dynamin, dynein, eEF-2, lamin B, Ulip2, vacuolar H^+^-ATPase (v-ATPase), synapsin and β-tubulin (Table 1). Of these identified proteins, v-ATPase, dynein and synapsins I and II are known PKA phosphorylation targets that are also involved in vesicle transport and release. Identification of dynamin and eEF-2 was unexpected, as they have not been classified as PKA targets, but regulate presynaptic vesicle transport/release and protein synthesis, respectively.

**Table 1:** Phospho-proteomic identification of targets induced following ethanol treatment in WT mice.

Spot Number	Protein Name	Spot Number	Protein Name
1	Dynamin	7	Lamin B
2	Elongation Factor 2	8	Ulip2
3	Cytoplasmic Dynein	9	Synapsin
	Intermediate Chain 1B		
4	Lamin B	10	No ID
5	Lamin B, Ulip2	11	Synapsin
6	Vacuolar H+-ATPase	12	β-Tubulin

Eleven proteins were identified as PKA targets in the cortex of WT mice following ethanol treatment. Spot number corresponds to location on 2D high resolution gel images presented in Figure 1.

### Immunoblot and Immunohistochemistry Analysis

**Figure 2:**
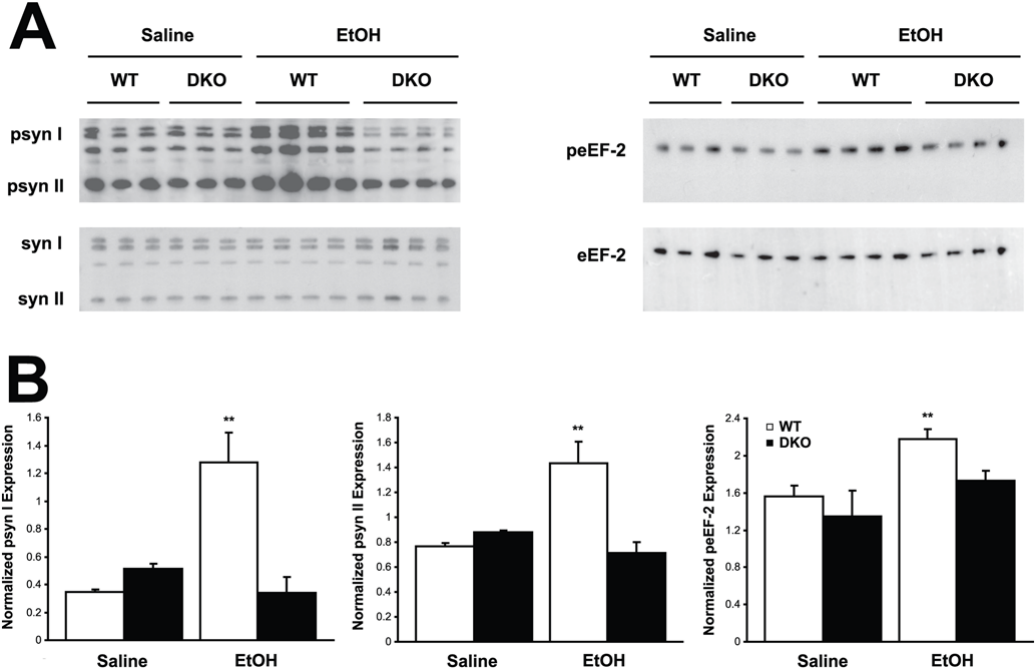
Ethanol-induced phosphorylation of synapsin I and II and eEF-2 is compromised in DKO hippocampus. (A) Immunoblot analysis of whole cell lysates from WT and DKO mice demonstrates increased expression of phosphorylated synapsin I and II (psyn I, II) and eEF-2 (peEF-2) in WT, but not DKO hippocampus following ethanol treatment compared to saline controls. (B) Quantification of phospho-synapsin I, II and phospho-eEF-2 expression normalized to total synapsin I, II (syn I, II) or eEF-2 expression, respectively. Ethanol significantly induced phosphorylation of synapsin I, II and eEF-2 in WT, but not DKO mice. (**, p<0.05 vs. WT saline).

**Figure 3:**
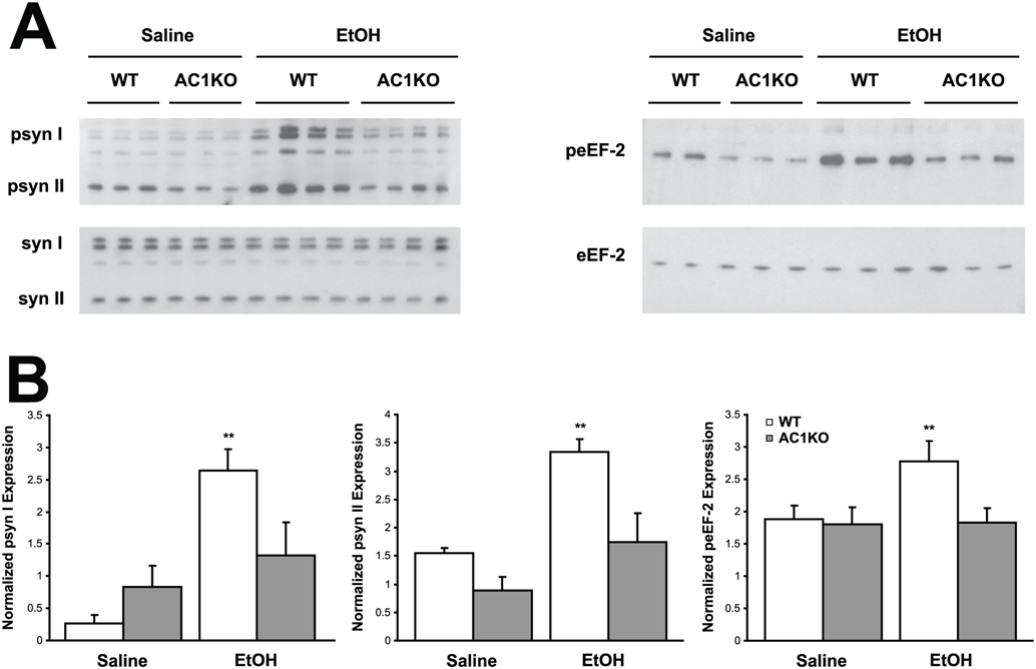
Ethanol-induced phosphorylation of synapsin I and II and eEF-2 is compromised in AC1KO hippocampus. (A) Immunoblot analysis of whole cell lysates from WT and AC1KO mice demonstrates increased expression of phosphorylated synapsin I and II (psyn I, II) and eEF-2 (peEF-2) in WT, but not AC1KO hippocampus following ethanol treatment compared to saline controls. (B) Quantification of phospho-synapsin I, II and phospho-eEF-2 expression normalized to total synapsin I, II (syn I, II) or eEF-2 expression, respectively. Ethanol significantly induced phosphorylation of synapsin I, II and eEF-2 in WT, but not AC1KO mice. (**, p<0.05 vs. WT saline).

**Figure 4:**
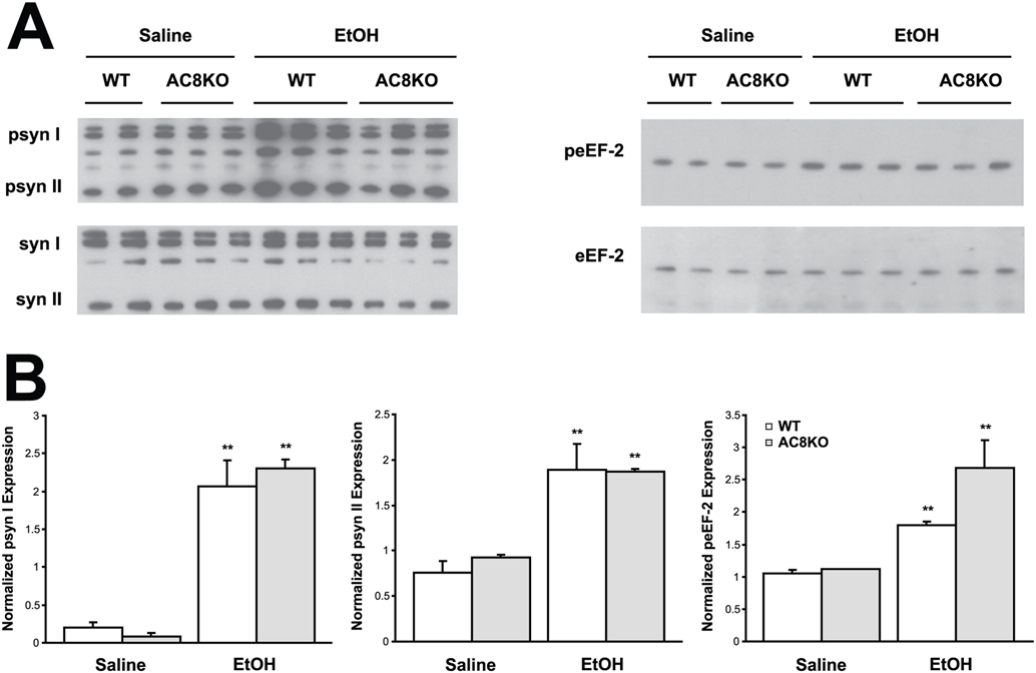
Ethanol-induced phosphorylation of synapsin I and II and eEF-2 is not compromised in AC8KO hippocampus. (A) Immunoblot analysis of whole cell lysates from WT and AC8KO mice demonstrates increased expression of phosphorylated synapsin I and II (psyn I, II) and eEF-2 (peEF-2) in WT and AC8KO hippocampus following ethanol treatment compared to saline controls. (B) Quantification of phospho-synapsin I, II and phospho-eEF-2 expression normalized to total synapsin I, II (syn I, II) or eEF-2 expression, respectively. Ethanol significantly induced phosphorylation of synapsin I, II and eEF-2 in WT and AC8KO mice. (**, p<0.05 vs. respective saline control).

**Figure 5:**
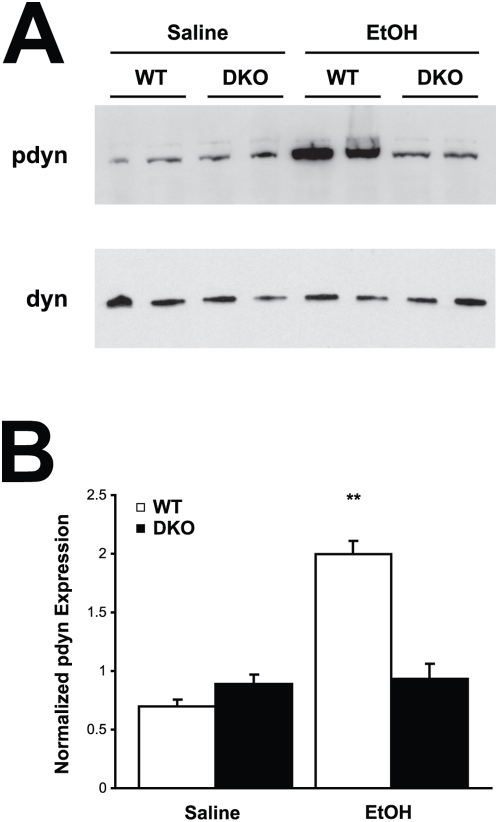
Ethanol-induced phosphorylation of dynamin is compromised in DKO hippocampus. (A) Immunoblot analysis of whole cell lysates from WT and DKO mice demonstrates increased expression of phosphorylated dynamin (pdyn) in WT, but not DKO hippocampus following ethanol treatment compared to saline controls. (B) Quantification of phospho-dynamin expression normalized to dynamin I/II. Ethanol significantly induced phosphorylation of dynamin in WT, but not DKO mice. (**, p<0.05 vs. WT saline).

Using immunoblot and immunohistochemistry techniques, impairments in synapsin phosphorylation were evaluated, as a representative presynaptic phospho-protein regulated by AC/PKA activity. Also, eEF-2 and dynamin phosphorylation was examined as representatives of the non-PKA substrate proteins identified. Whole cell hippocampal and cortical lysates were collected from WT, DKO, AC1KO and AC8KO mice 45 min following ethanol exposure. Phosphorylation of synapsins I and II was significantly increased in the hippocampus (Figure 2) and cortex (data not shown) of WT mice after ethanol treatment. These robust increases in phosphorylation of synapsin I and II were not observed in either brain region of DKO or AC1KO mice (Figures 2 and 3). Levels of phospho-synapsin I and II in ethanol-treated AC1KO and DKO mice were unchanged from those of saline-treated WT and DKO controls (Figures 2 and 3). The phosphorylation of synapsin in AC8KO mice resembled that of WT mice in both the hippocampus and cortex following ethanol exposure (Figure 4, data not shown). Similarly, while WT and AC8KO mice demonstrated significant increases in eEF-2 phosphorylation in the hippocampus (Figure 4) and cortex (data not shown) following ethanol treatment compared to controls, levels of phospho-eEF-2 in DKO and AC1KO mice were unchanged from saline-treated controls (Figures 2 and 3). Likewise, dynamin phosphorylation in WT and DKO hippocampal extracts was examined following acute ethanol treatment (Figure 5). Phosphorylation of dynamin was significantly increased in WT mice compared to saline-treated controls, while DKO mice demonstrated no change from controls in levels of phospho-dynamin following ethanol exposure (Figure 5).

**Figure 6:**
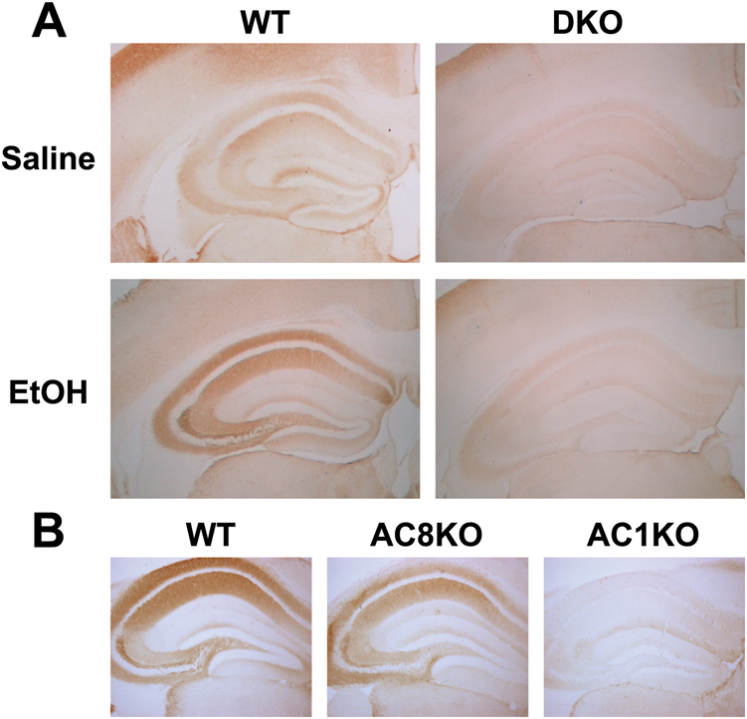
Immunohistochemical detection of phospho-synapsin protein following ethanol treatment in WT and ACKO mice. (A) Representative coronal sections at 20× magnification demonstrate robust induction of phospho-synapsin in the cortex and hippocampus of ethanol-treated WT mice compared to saline controls. DKO mice demonstrate no induction of phospho-synapsin in either brain region following ethanol treatment compared to saline controls. (B) Representative coronal sections at 40× magnification demonstrate robust induction of phospho-synapsin in the hippocampus of ethanol-treated WT and AC8KO mice. In contrast, AC1KO mice impaired induction of phospho-synapsin in following ethanol treatment compared to WT and AC8KO mice.

Brains of AC1KO, AC8KO, DKO, and WT mice were harvested at 45 min following acute ethanol exposure. Immunohistochemistry for phospho-synapsin revealed a dramatic increase throughout the hippocampus and cortex of WT and AC8KO mice (Figure 6). Within the neuron phospho-synapsin was localized to cellular projections but not in cell bodies, as expected. AC1KO and DKO mice exhibited little induction of synapsin phosphorylation in either the cortex or hippocampus, in concordance with immunoblot results (Figure 6).

### AC1/AC8 deficient mice demonstrate a reduced pool of functional recycling vesicles

**Figure 7:**
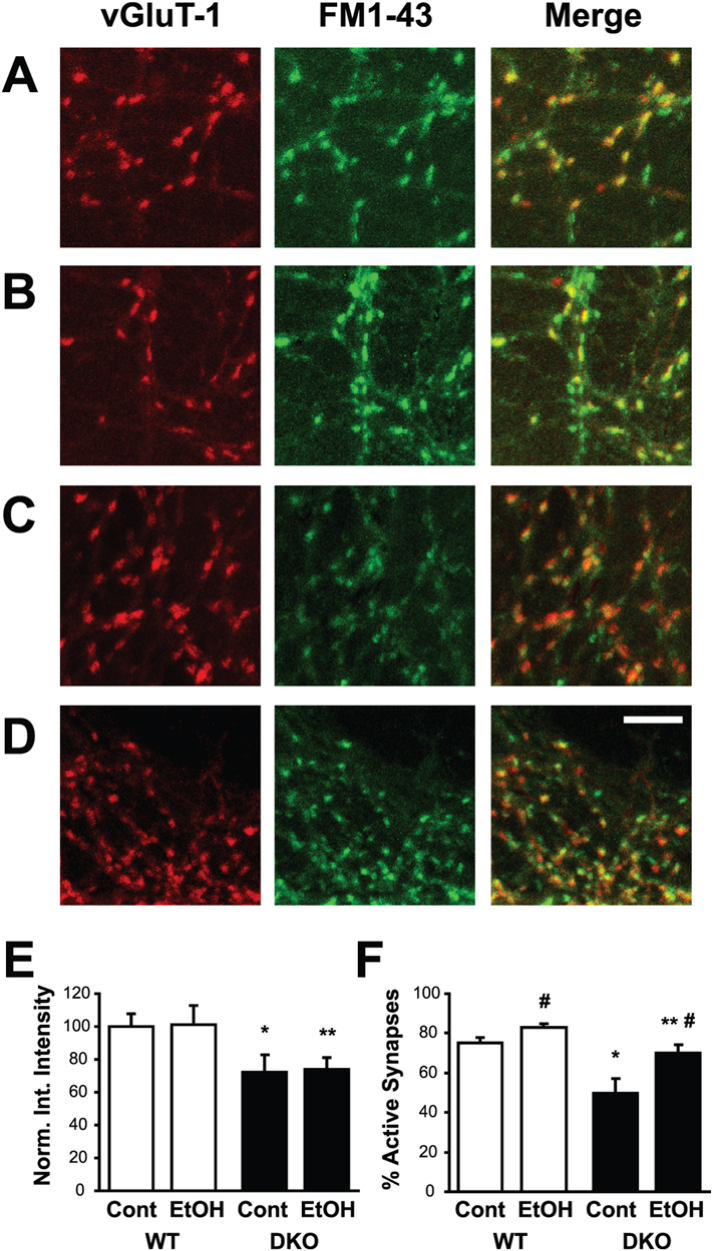
Recycling pool size measured with FM1-43FX labeling in WT and DKO neurons following ethanol exposure. Labelling with FM1-43 (green) identifies active synapses and *post hoc* vGluT-1 immunofluorescence (red) identifies glutamatergic synapses in WT control (A), WT ethanol-treated (B), DKO control (C) and DKO ethanol-treated (D) hippocampal cultures. Merged image illustrates presence of active glutamatergic synapses (FM1-43 positive, vGluT-1 positive, yellow) and inactive (FM1-43 negative, vGluT-1 positive, red) glutamatergic synapses. Scalebar represents 10 µm. (E) Summary of the normalized integrated intensity from fields obtained in blinded analyses. (*, p<0.03 vs. WT; **, p<0.04 vs. WT+ethanol). (F) Summary of the percentage of active synapses from fields obtained in blinded analyses in WT and DKO cultures. (*, p<0.007 vs. WT; **, p<0.045 vs. WT+ethanol; #, p<0.05 vs. respective control). For E and F, n = 15–20.

In order to determine whether the presynaptic alterations we observed had an effect on synaptic vesicle cycling, we assessed uptake of the styryl dye, FM1-43, in dissociated hippocampal neurons (Figure 7). Neurons obtained from WT and DKO animals were incubated in the presence or absence of 100 mM ethanol for 30 minutes. FM1-43 labeling was then assessed during a depolarizing challenge designed to stimulate exo/endocytosis of the entire pool of recycling-competent vesicles [16]. FM1-43 uptake was reduced at individual glutamate synapses in DKO compared to uptake in WT neurons, although ethanol exposure did not change FM1-43 uptake from the control condition in either genotype (Figure 7E). In addition, the percent of glutamate (vGluT-1-positive) synapses that labeled with FM1-43, defined as the percent of active synapses, was lower in DKO neurons compared to WT neurons (Figure 7F). In both WT and DKO neurons, ethanol treatment significantly increased the percentage of active synapses (p<0.044 for WT and p<0.02 for DKO). These data indicate that presynaptic function is compromised in DKO neurons. Therefore, although synapses from DKO neurons appeared to respond to ethanol in a similar manner as synapses from WT neurons, DKO neurons responded from an altered basal level of synaptic activity. Furthermore, the percentage of active synapses following ethanol exposure in DKO neurons was significantly reduced compared to ethanol-treated WT neurons.

## Discussion

The present study reveals a role for AC1 and AC8 in mediating the homeostatic response to ethanol-induced activity blockade. Phosphoproteomic analyses identified targets primarily localized to presynaptic vesicle transport machinery that were phosphorylated following acute ethanol exposure in the brains of WT mice. Changes in phospho-synapsin I and II, phospho-eEF-2 and phospho-dynamin were further validated with immunoblot and immunohistochemical techniques and found to be significantly reduced in the cortex and hippocampus of DKO and in some cases, AC1KO mice compared to WT controls. Reduced integrated intensity of FM1-43 uptake in DKO hippocampal neurons demonstrated a decreased number of functional recycling vesicles and a reduced number of active terminals, which likely contributes to the impaired presynaptic response observed in DKO mice.

Ethanol has been shown to modulate several aspects of the cAMP/PKA signaling cascade. PKA activation and nuclear translocation is increased after ethanol exposure and is accompanied by cAMP response element (CRE) binding protein (CREB) phosphorylation, CREB binding protein (CBP) phosphorylation and CRE-mediated gene transcription [17]–[20]. Our previous studies have demonstrated a specific role for AC1 and AC8 in mediating phosphorylation of a discrete subset of PKA targets following acute ethanol treatment in the adult mouse brain [1].

In the present study we have identified phospho-protein PKA targets that were phosphorylated in the adult brain following acute ethanol exposure, including v-ATPase, dynein and synapsin. Additional proteins phosphorylated after ethanol treatment included dynamin and eEF-2. Dynamin, a microtubule associated motor enzyme, supports the transport and/or budding of vesicles along microtubules. In addition, the expression of dynamin I is neuron-specific and enriched in nerve terminals where it is required for synaptic vesicle fission from the plasma membrane [21]. The phosphorylation status of dynamin I regulates synaptic vesicle endocytosis [21], suggesting that impairment of this process by loss of AC activity may impact vesicle recycling, a process essential to activity-dependent neurotransmission [22], [23]. This is supported by data from dynamin I-deficient mice that exhibit impaired stimulation-dependent synaptic vesicle recycling [24]. Ethanol has been demonstrated to reduce vesicle transport in hepatocytes by a mechanism that may involve a change in dynamin function [25]; however, our data are the first to suggest a role for dynamin in the brain following ethanol exposure. As it is primarily phosphorylated by cyclin-dependent kinase 5 (cdk5), the detection of dynamin phosphorylation suggests the possibility of cross-reactivity of the phospho-PKA-substrate antibody to cdk5 phosphorylation sites.

The ATP-dependent proton pump, v-ATPase, acidifies intracellular compartments, including synaptic vesicles [26]. PKA has been demonstrated to phosphorylate the V_1_ subunit, a molecule that controls v-ATPase disassembly/reassembly dynamics, thereby mediating v-ATPase activity [27]. This suggests that alterations in transporter phosphorylation may lead to changes in transmitter packaging/release, ultimately resulting in changes in synaptic efficacy. In this case, postsynaptic currents would be affected while FM1-43 uptake into vesicles may remain unchanged [28]. Previous studies have demonstrated that deletion of genes regulating hydrogen-transporting ATPase activity and vacuole acidification conferred cosensitivity to several alcohols, including ethanol, in a genome-wide screen of yeast deletion mutants suggesting that v-ATPase function is required for alcohol tolerance [29].

Elongation factor-2, a key component in peptide-chain elongation, has recently been implicated in the mechanism by which ethanol inhibits protein synthesis in skeletal myocytes [30]. While eEF-2 has not been demonstrated to be a direct target of PKA, eEF-2 kinase is phosphorylated in a calcium-calmodulin and PKA-dependent manner [31], [32]. This suggests that eEF-2 phosphorylation is a mechanism by which increases in intracellular calcium concentrations, a known consequence of ethanol exposure, can modulate the rate of protein synthesis.

Synapsins have been shown to bind synaptic vesicles to the cytoskeleton, segregating them to form a reserve pool and an active pool [33], [34]. Synapsins are phosphorylated by PKA at serine 9, which inhibits their binding to phospholipids and dissociates them from synaptic vesicles [35]. Previous reports have documented ethanol's ability to increase clustering of synapsin in vitro [36], [37]. These data are in agreement with the observation that prolonged synaptic inactivity increases the size of the presynaptic active zone, the number of docked vesicles and the total number of vesicles [38]. Increased phosphorylation of dynamin, v-ATPase and synapsin suggest that presynaptic vesicle transport and release are critical to the neuronal response to ethanol exposure, while phosphorylation of v-ATPase and eEF-2 are associated with cell maintenance and function in the presence of ethanol.

The localization of AC1 in cortical and hippocampal regions strategically poise it to modulate cAMP-dependent activation of PKA following ethanol treatment. AC1 is strongly expressed in axons and terminals of the mossy fiber pathway and in subcellular synaptic fractions that are enriched with synaptophysin, a presynaptic vesicle protein [7]. This places AC1 proximal to the robust expression of phospho-synapsin in the CA3 region of the hippocampus after ethanol exposure and associates AC1 with presynaptic vesicle regulation. In the present study, AC1KO mice exhibited impairments in synapsin and eEF-2 phosphorylation while AC8KO mice did not. These data are in agreement with previous studies from our laboratory demonstrating that both AC1KO and DKO mice exhibit enhanced sensitivity to the sedating effects of ethanol as compared to WT mice, whereas AC8KO mice do not [1], underscoring the importance of AC1 in the initiation of presynaptic homeostatic events following activity blockade. Although AC1 has previously been associated with postsynaptic mechanisms, these data emphasize the importance of AC1 in presynaptic vesicle release mechanisms and are supported by the recent findings in *barrelless* mice containing a loss-of-function mutation in the AC1 gene. *Barrelless* mice demonstrate deficits in neurotransmitter release attributed to reductions in AC1-dependent PKA phosphorylation of RIMs [10]. Likewise, PKA phosphorylation of synapsins is reduced in *barrelless* mice suggesting impairments in synaptic vesicle mobilization [10], [39]. Together, these data support the hypothesis that interventions that alter levels of neuronal activity, such as ethanol exposure, result in counteractive homeostatic mechanisms against such alterations.

Here we demonstrate that WT, but not DKO, mice respond to acute ethanol exposure with an increase in synapsin phosphorylation. While ethanol exposure does not affect the net size of the recycling vesicle pool in WT or DKO mice, phosphorylation status of synapsin has been shown to drive the rate of its dispersion, thereby regulating the dynamics of vesicle pool turnover [39]. In this way, synapsin regulates the rate of synaptic vesicle mobilization and release in a phosphorylation-state-dependent manner [39]. Together, these data suggest that while recycling vesicle pool size is unchanged in WT and DKO mice after ethanol treatment, an increase in vesicle cycling rate may drive the reactivation of WT neurons, a mechanism that is impaired in DKO mice due to impaired synapsin phosphorylation. Combined with the decreased integrated intensity of FM1-43 labeling observed in DKO neurons, which represents a reduction in the pool size of recycling vesicles, the lack of synapsin phosphorylation may contribute to a slower rate of vesicle recycling and therefore a disruption in neuronal reactivation following activity blockade by ethanol in DKO mice. Similarly, phosphorylation of dynamin has been suggested to regulate the rate of synaptic vesicle endocytosis (SVE) [40]. While alterations in the rate of SVE may not be manifested as a change in the recycling vesicle pool size, reactivation of neurons from ethanol-induced activity blockade may depend on the efficiency of neurotransmission. This is supported by data from dynamin I-deficient mice that exhibit impaired stimulation-dependent synaptic vesicle recycling and the requirement of dynamin for SVE during periods of high neuronal activity [24]. These data suggest that a threshold of vesicle recycling is required for the appropriate neuronal response to activity blockade. As evidenced by the baseline reduction in the percentage of active synapses in DKO compared to WT mice and the decrease in the percentage of active synapses after ethanol exposure in DKO neurons compared to WT, AC1 and AC8 contribute to maintaining a critical threshold of synaptic activity both in the presence or absence of ethanol.

Together, these data demonstrate a pivotal role for the calcium-stimulated ACs, mainly AC1, in the initiation of a presynaptic response to ethanol-mediated neuronal inhibition. Further identification of PKA targets uniquely regulated by AC1 and AC8 will provide additional insight into the mechanisms of the neuronal response to the inhibitory effects of ethanol.

## Materials and Methods

### Animal Husbandry

All mice were backcrossed a minimum of ten generations to wild type C57BL/6 (WT) mice from The Jackson Laboratory (Bar Harbor, ME). To generate mice for these experiments, we used progeny of homozygous mutants (AC1KO, AC8KO or AC1/8KO) and WT mice from The Jackson Laboratory bred in our colony. Mice were maintained on a 12 hr light/dark schedule with *ad libitum* access to food and water. All experiments were performed using male mice between 2 and 4 months of age. All mouse protocols were in accordance with the National Institutes of Health guidelines and were approved by the Animal Care and Use Committee of Washington University School of Medicine.

### 2-Dimensional High Resolution Gel Electrophoresis

For detailed 2-Dimensional High Resolution Gel Electrophoresis, Protein Identification of Gel Features and Matrix-assisted Laser Desorption Ionization (MALDI) mass spectrometry methods, see Bredemeyer, et al [41]. Briefly, forty-five minutes following an intraperitoneal injection of ethanol to achieve a dose of 4.0 g/kg, WT mice were killed by CO_2_ inhalation and cortices were removed rapidly and frozen in liquid nitrogen. Tissues were homogenized in a buffer containing 4% (w/v) 3-[(3-cholamido-propyl) dimethylammonio]-1-propanesulfonate, 2 M thiourea, 7 M urea, and 30 mM Tris, pH 8.5. One tablet of Complete protease inhibitor mixture (Roche Products, Indianapolis. IN) was added to 50 mL of lysis buffer. After homogenization, samples were centrifuged at 8000×g for 10 min and the supernatants collected. Protein concentrations were determined using the 2D-Quant kit (Amersham Biosciences, Piscataway, NJ). First-dimension isoelectric focusing was performed on immobilized pH gradient strips (24 cm; pH 3.0–10.0, nonlinear) in an Ettan IPGphor system (GE Healthcare). Second-dimension separation was performed on 10% isocratic SDS/PAGE gels (20×24 cm). Anti-phospho-PKA substrate antibody (Cell Signaling Technology, Beverly, MA) was used to detect phospho-proteins (1∶1000). Images were acquired on a Typhoon 9400 scanner (GE Healthcare) and relative quantification of matched gel features was performed by using Decyder DIA and BVA software (GE Healthcare).

### Protein Immunoblot Analysis

Forty-five minutes following an intraperitoneal injection of saline or ethanol to achieve a dose of 4.0 g/kg, mice (n = 4–6 per treatment/genotype) were killed by CO_2_ inhalation and brains were removed rapidly. Subregions were dissected and frozen in liquid nitrogen. Brains were homogenized in a buffer containing 4% (w/v) 3-[(3-cholamido-propyl)dimethylammonio]-1-propanesulfonate, 2 M thiourea, 7 M urea, and 30 mM Tris, pH 8.5. One tablet of Complete protease inhibitor mixture (Roche Products, Indianapolis. IN) was added to 50 mL of lysis buffer. After homogenization, samples were centrifuged at 8000×g for 10 min and the supernatants collected. Protein concentrations were determined using the 2D-Quant kit (Amersham Biosciences, Piscataway, NJ). Equal amounts of protein were submitted to 4–12% SDS-PAGE and transferred to nitrocellulose membrane. Membranes were probed with primary antibodies at the following concentrations: anti-phosphorylated synapsin, anti-synapsin, anti-phosphorylated eEF-2, anti-eEF-2, anti-dynamin I/II, 1∶1000 (Cell Signaling Technology, Beverly, MA) and anti-phosphorylated dynamin (pSer778), 1∶1000 (Sigma, St. Louis, MO). Antibodies were detected using HRP-conjugated goat anti-rabbit or donkey anti-sheep secondary antibodies and signals were visualized using chemiluminescence (SuperSignal West Dura kit; Pierce, Rockford, IL). Densitometric analysis was performed using NIH Image Software. For each sample, phosphoprotein signals were normalized to total protein signals and averaged within groups.

### Immunohistochemistry

Forty-five minutes following an intraperitoneal injection of saline or ethanol to achieve a dose of 4.0 g/kg, mice (n = 4–6 per treatment/genotype) were killed by CO_2_ inhalation and brains were removed rapidly. Brains were dissected into 1 mm thick coronal slices and immersion fixed in 4% paraformaldehyde in 0.1 M PBS overnight at 4°C. Slices were cryoprotected in 30% sucrose for 3 days, embedded in mounting medium, and stored at −80°C. Frozen tissues were cut into 40 µm slices and stored free-floating in 1× PBS/0.1% NaN_3_ at 4°C until use.

Free floating sections were quenched of endogenous peroxidases with 0.3% H_2_O_2_/0.75% Triton X-100 for 1 h, washed in 1× PBS and blocked with 1% normal goat serum/10% fish gel/0.6% nonfat dry milk (blocking solution A) for 1 h. Sections were incubated in rabbit anti-phospho-synapsin antibody (1∶500, Cell Signaling in blocking solution A) overnight at 4°C followed by incubation in blocking solution A for 1 h. Following treatment with biotinylated goat anti-rabbit secondary antibody (Vector Laboratories) at 1∶500 for 1 h, sections were blocked again as described. Biotin was detected with an ABC kit (Vector Laboratories) and visualized by incubation in DAB for 3 min. Sections were slide-mounted, dehydrated and preserved using Permount mounting medium. All images were obtained using matched settings between genotypes and treatments on an Olympus BX60 microscope equipped with Axiovision software. Images were prepared using Adobe Photoshop software.

### Cell culture

Dissected postnatal (P0–P1) mouse hippocampi were incubated with papain, and then mechanically dissociated and plated at 2000 cells/mm^2^. Plating medium consisted of Eagle's medium (Invitrogen, Gaithersburg, MD) supplemented with heat-inactivated horse serum (5%), fetal bovine serum (5%), 17 mM glucose, 400 µM glutamine, 50 U/ml penicillin, and 50 µg/ml streptomycin. Cultures were maintained at 37°C in a humidified incubator with 5%CO_2_/95% air. 6.7 µM cytosine arabinoside was added 3–4 days after plating to inhibit cell division. At 4–5 days after plating, a ½-volume medium replacement was conducted using Neurobasal medium (Invitrogen) plus B27 supplement.

Experiments were conducted at 11–14 DIV from WT and DKO cultures that had been plated separately on the same day. Ethanol was added directly to the culture medium to initiate exposure. Cultures were then transferred to a closed, humidified chamber containing ethanol at the same concentration as the culture medium (100 mM). Control dishes were placed in an identical chamber containing water. Cultures were maintained in a 37°C incubator with a mixture of 5% CO_2_/95% air for 30 min.

### FM1-43 imaging and immunocytochemistry

Hippocampal cultures plated on cover slips were used for all imaging experiments. Active synapses were labeled with a 2 min application of 10 µM FM1-43FX (Molecular Probes, Eugene, OR) and 45 mM K^+^ in a saline solution containing (in mM): 138 NaCl, 4 KCl, 2 CaCl_2_, 1 MgCl_2_, 10 glucose, 10 HEPES, 0.025 D-APV, and 0.001 NBQX (pH 7.25). Cultures were washed for 10 s with saline containing 500 µM Advasep-7 (CyDex, Inc., Overland Park, KS), and then in saline alone for 10 min. Cultures were fixed in 4% paraformaldehyde/0.2% glutaraldehyde in PBS for 10 min.

After fixation, cells were washed with PBS and exposed to blocking solution (10% normal goat serum/0.05% Triton X-100 in PBS) for 15 min. Cells were incubated with vGluT-1 antibody (Chemicon, Temecula, CA) diluted at 1∶2500 in blocking solution for 4 hr. After primary antibody incubation, cells were washed with PBS and then incubated with Cy3-conjugated anti-guinea pig antibody (1∶200 in blocking solution; Chemicon) for 30 min. Cover slips were then washed with PBS and mounted with Fluoromount-G (Southern Biotechnology Associates, Birmingham, AL).

Confocal imaging was performed using a 60× objective (1.4 N.A.), a C1 scanning confocal laser attached to an inverted Eclipse TE300 microscope (Nikon Instruments, Melville, NY), and Z-C1 software (Nikon). A naïve observer acquired images of representative fields in z-stack using alternating excitation by the 488 nm and 543 nm laser lines. Gain settings, dwell time, field of view size, and z-stack parameters were kept constant for all images within an experiment. Monochrome images were converted into projected images and analyzed using Metamorph software (Universal Imaging, Downingtown, PA). Ten puncta per field and 5 fields per condition were analyzed for each experiment.

### Statistical analysis

Two-way ANOVA followed by Bonferonni's post hoc tests, where appropriate, were done to evaluate statistical significance. A p value of less than 0.05 was considered to be statistically significant. Results are displayed as mean±s.e.m.
